# Navigating synergies vs. trade-offs between climate change mitigation and biodiversity conservation

**DOI:** 10.1038/s44185-025-00092-8

**Published:** 2025-06-03

**Authors:** Shermin de Silva, Natalie Jacewicz, Karen Kovaka, Kristy Ferraro, Craig Callender, Dale Jamieson, Aseem Prakash

**Affiliations:** 1 Department of Ecology, Behavior and Evolution, UC San Diego, La Jolla, CA USA; 2 Trunks & Leaves Inc., Pittsfield, MA USA; 3 School of Law, University of San Diego, San Diego, CA USA; 4 Department of Philosophy, UC San Diego, La Jolla, CA USA; 5 Institute for Practical Ethics, UC San Diego, La Jolla, CA USA; 6 School of the Environment, Yale University, New Haven, CT USA; 7 Department of Biology, Memorial University, St. Johns, NL Canada; 8 Department of Environmental Studies, New York University, New York, NY USA; 9 Department of Political Science, University of Washington, Seattle, WA USA

**Keywords:** Ethics, Climate-change policy, Biodiversity

## Abstract

Synergies between mitigating climate change and conserving biodiversity are often emphasized in public discourse and policy, but there can be trade-offs between these aims. Where trade-offs are evident, cost-benefit analysis (CBA) has emerged as a dominant approach to resolving them. We highlight limitations of this approach and propose that creating enviro-ethics committees using principles of collaborative governance would provide a practical mechanism for transparently grappling with trade-offs at various levels.

## Introduction

Climate change and biodiversity are among the foremost environmental challenges facing modern society. Although climate change impacts may seem more noticeable and garner more public attention^1,2^, both command a significant portion of research and policy energy^3,4^. Therefore, tackling these challenges fairly and efficiently is crucial. However, many climate and biodiversity policies were historically developed independently. In the 1990s, this siloed approach was institutionalized at the international level with the establishment of independent United Nations conventions on climate change (United Nations Framework Convention on Climate Change, or UNFCCC) and biodiversity (Convention on Biological Diversity, or CBD). This separation of issues is reflected in national legal structures as well. For example, in the United States, the task of combating climate change falls primarily to energy and pollution-regulating agencies, such as the Environmental Protection Agency and the Department of Transportation, whereas protecting endangered species is under the purview of the US Fish and Wildlife Service and the National Oceanic and Atmospheric Administration. Court rulings also distinguish agencies’ obligations toward wildlife conservation from those addressing climate change. For example, the District Court of Columbia ruled that the US Fish and Wildlife Service has no obligation to address climate change as part of its efforts to protect threatened polar bears^5^.

At the global level, the siloing of climate change and biodiversity is slowly changing, with policies intended to simultaneously advance both goals^6–8^. Claims regarding synergies (win-wins) between climate change mitigation and biodiversity conservation feature prominently in emerging research and policy agendas^6,9,10^. For example, restoring degraded ecosystems also captures carbon and enhances biodiversity^3^. Policy frameworks such as “nature-based solutions” attempt to address the challenges of climate change and biodiversity loss simultaneously^11,12^. The European Union founded the European Climate, Environment, and Infrastructure Executive Agency in 2021 to implement both climate change and ecosystem-based projects. In the United States, the Biden administration released an executive order to “Tackl[e] the Climate Crisis at Home and Abroad,” which incorporates the goal to conserve 30 percent of the nation’s lands and waters by 2030, reflecting a unified approach to combat climate change and biodiversity loss^13^. These initiatives directly reflected goals adopted by the international community under the CBD (specifically, Target 3 of the Kunming-Montreal Global Biodiversity Framework or KMGBF), even though the United States is not a party to the convention.

The prospects for win-wins for both climate and biodiversity have also been increasingly emphasized in research. A recent systematic review of nature-based solutions for climate adaptation found co-benefits for ecosystem health (in terms of biodiversity protection) in 88% of cases^11^. The authors contend that policymakers can ensure that “nature-based climate policy always supports ecosystem health.” Similarly, other researchers have claimed that interventions to conserve biodiversity “generally benefit” climate goals^14^ and that very few climate mitigation and adaptation practices have negative impacts on biodiversity^15^. However, as we caution below, win-win outcomes are not a given, and in fact appear to be more unlikely with increasingly complex biological and sociopolitical systems^16^.

**Fig. 1:**
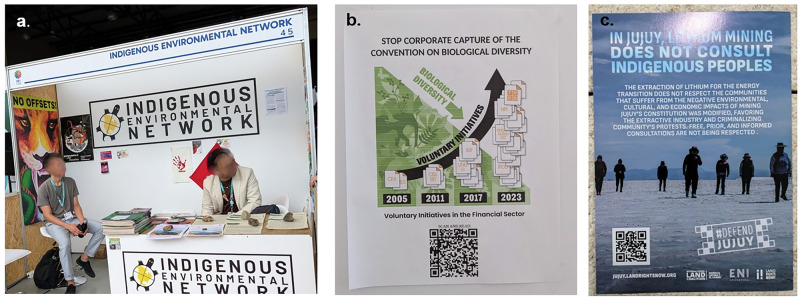
Tensions from trade-offs at CBD COP-16. **a**, **b** Opposition to biodiversity offsets and other financial mechanisms that have cost-benefit analyses embedded within them was prevalent at CBD COP-16. **c** A flyer protesting the violation of indigenous land rights for lithium mining. Images: S. de Silva.

Despite the rhetoric, tensions between climate and biodiversity goals have been evident at the Conferences of the Parties to the UN Convention on Biological Diversity (CBD). At the 2024 COP-16 meeting, national delegates emphasized the need to simultaneously address both climate change and biodiversity loss, with text underscoring the relationship between them (Decision 16/22), mentioning the word “synergies” no less than seven times in six pages. On the other hand, mentions of trade-offs in draft text were omitted from the final text, aside from oblique statements to “avoid or, if not possible, minimize the negative impacts of climate actions on biodiversity and ecosystem integrity… in particular for indigenous peoples and local communities and relevant stakeholders that directly depend on biodiversity.” The decision also proposes a joint work program among the two Rio conventions to facilitate alignment. A full-day side event focused on how to equitably meet target 3 of the KGBF. Meanwhile, in a parallel side event, leaders from the Maasai community voiced their desire for the removal of protected areas from traditional lands, denouncing carbon credits as well as ecotourism as “business ventures.” Indigenous participants throughout the event emphasized the need for free, prior, and informed consent for activities conducted on their traditional territories, which are threatened by activities such as mining for critical minerals (Fig. 1). Speaking at COP-16, the Executive Secretary of the UN Convention to Combat Desertification (UNCCD), Ibrahim Thiaw, referred to the term “synergy” as the result of “scientific jargon and diplomatic barbarism,” to the effect that “no one knows what it means”—but asserted that surely reversing desertification was one means of achieving it.

The possibility of synergies does not imply an absence of trade-offs. Trade-offs clearly occur in numerous cases, notably when resources (e.g., time, money, land, water) are limited, as they usually are. For instance, large-scale solar and wind farms in ecologically sensitive areas can harm vulnerable species^10^. The mining of rare earth elements raises a host of both ecological and social concerns^17^. Afforestation for carbon sequestration can threaten grassland biomes^18^. Conversely, some projects that are intended to support biodiversity conservation could contribute to climate change, such as the carbon-intensive travel that often accompanies “ecotourism,” or maintaining grassland diversity through grazing.

With this paper, we hope to move the conversation forward in two ways by: (1) showing how cost-benefit analyses are inadequate for reconciling trade-offs, and (2) developing a new avenue to grapple with inescapable tensions between addressing climate change and protecting biodiversity from a standpoint of environmental justice and equity. Cost-benefit analysis (CBA) is a widely employed tool to assess policy trade-offs that promises to impart objectivity in balancing technical and quantitative environmental trade-offs. However, we explain why it does not present the whole story when weighing the effects of climate change against threats to biodiversity. We then suggest an alternative approach: establishing environmental ethics committees to deliberate and advise on trade-offs that adequately weigh differing perspectives and priorities, especially of marginalized groups^19,20^. Such committees can help to ensure input from a wide range of stakeholders in assessing progress on climate change and biodiversity *across* initiatives. Instead of a technocratic, one-size-fits-all approach, such committees could come up with context-specific criteria to assess the broad range of stakeholder concerns.

### Climate-biodiversity trade-offs and the mismatch of cost-benefit analysis

At first blush, CBA may seem well-suited to weigh a project’s beneficial effects on climate change against its harmful effects on biodiversity, or vice versa (e.g., the number of tons of carbon removed or added, the number of wetland acres or species saved or developed). To do so, it is often assumed that a thorough CBA can accurately and objectively put a monetary value on the gains and losses under various policies^21^. For example, Executive Order 12866 requires US federal agencies to conduct cost-benefit analyses of the impact of many regulations^22^. As long as benefits exceed costs, the project can be deemed a net ‘win.’ CBA also relies on the rationale that any required compensation (hypothetical or real) where “winners” compensate the “losers” is morally just and politically acceptable^23^. It is a popular policy approach, in part due to the growing ability to monetize ecosystem services, functions, and other benefits. CBA features prominently in evaluating policy options with multiple, quantifiable benefits and costs, such as in evaluating the public health impact of greenhouse gas emissions standards for power plants or pollution policy generally^24,25^. Even when CBA does not fully determine which policies are adopted, its use in the decision-making process still has far-reaching implications for how the advantages of different options are framed and evaluated. For example, Millennium Ecosystem Assessment’s way of differentiating ecosystem services has been criticized for its lack of fit with CBA procedures^26^.

Notwithstanding these advantages of CBA, its challenges are well-known in general^23^ as well as specifically for the environment and biodiversity^12,25,27^. We briefly outline several reasons to be skeptical about CBA’s ability to resolve trade-offs between climate change mitigation and biodiversity conservation goals.

#### Climate versus biodiversity indicators

Atmospheric greenhouse gases (GHGs) can be quantified with standardized measurement techniques. Biodiversity, by contrast, enjoys no clear consensus on indicators. Nearly 100 indicators have been proposed to meet the Convention on Biological Diversity’s 2020 targets, including trends in genetic diversity, species population, and extinction risk, as well as the extent of different kinds of forests^28,29^. Yet none act as the primary metric of progress. As a result, efforts to compare the effects of a particular policy on climate change and biodiversity may result in well-quantified climate outcomes but an incomplete or unrepresentative quantification of biodiversity impacts.

#### Localized vs. distributional effects

Greenhouse gases circulate freely in the atmosphere; a ton of CO_2_ is a ton of CO_2_ no matter where it is emitted. By contrast, biodiversity metrics typically represent a highly localized context: protecting taxon A in area X has different implications for biodiversity than protecting taxon B in area Y. Further, biodiversity impacts tend to disproportionately affect local communities (for better or worse), while carbon impacts have the potential to ripple through various scales. For example, a community that conducts ecotourism enjoys concentrated biodiversity benefits, while the negative climate externalities are distributed across the globe. Conversely, a community that opposes a renewable energy project may do so based on harm to local ecosystems, while the climate benefits are globally distributed.

#### Fungibility versus non-fungibility

Comparing fungible and non-fungible goods is a classic problem for CBA. Biodiversity, unlike CO_2_, is not fungible. Due to circulation in the atmosphere, a reduction in CO_2_ from anywhere is typically as good as from anywhere else. That isn’t true for biodiversity, where (say) a wetland in one location may contain more species than a wetland in another. In addition to lacking a consensus indicator for biodiversity, we also lack a consensus indicator jointly covering both biodiversity and climate. One practical consequence is that carbon offset markets exist and are well-developed, whereas biodiversity offset markets are controversial (Fig. 1), and joint carbon-biodiversity offset markets do not yet exist.

There are some ways to compare non-fungible items in CBA. For example, the value of a life is quantified via consumer preferences for saving one “statistical” life^30^. But without an actual market in human lives, these valuations are often very indirect and yield disputable values. We can expect the same here. In a cost-benefit analysis of whether to place a solar farm in a critical desert habitat, the value of the solar farm is relatively easy to determine because it is fungible and part of a market. The habitat is not, and thus prone to over- or under-valuing. This asymmetry in ease of valuation is not, in principle, insoluble, nor always the case, but generally exacerbates already difficult challenges for CBA.

#### Somewhat predictable socioeconomic risks versus contestable risks

Although climate science is tremendously complex, it rests on basic physical chemistry. These generalizations enable scientists to link GHG levels to changes in physical variables, such as temperature. Despite geopolitical uncertainties, the predicted impacts of climate change on humans can be readily quantified, exemplified by concepts such as the “social cost of carbon”^31–33^. There is no such analog for biodiversity. Ecology is often at a disadvantage relative to climate science, both with respect to predicting impacts and evaluating risk. Consider deep-sea mining for critical minerals, often presented as necessary for decarbonization. Our lack of knowledge about seabed ecosystems makes it impossible to say what kinds of biodiversity loss such mining will cause. The value of these ecosystems, about which we know very little, is contestable^34^. Perhaps most challenging are cases when the monetary and non-monetary values of different stakeholders clash^35,36^, which might render CBAs altogether inappropriate.

We are not suggesting that CBA is always impractical, nor are these trade-offs unique to the domains of climate impacts and biodiversity loss. However, the particular task of trading off biodiversity against climate change implicates a broad range of value judgments that are difficult to resolve through CBA. Additional approaches to face trade-offs are required.

### A path forward

Bioethics and collaborative governance practices offer alternative paradigms^37–40^. The origins of bioethics committees can be traced back to at least the end of the Second World War, when scientists and medical practitioners in Europe were tried for war crimes^37^. In 1974, the United States created the National Commission for the Protection of Human Subjects of Biomedical and Behavioral Research as part of the National Research Act. Such national commissions continued in the United States until 2017. Hospital ethics began to be formed in the 1980s, and by 1986, recipients of federal funds were required to also establish institutional animal care and use (IACUC) committees. Likewise, Institutional Review Boards (IRB) evaluate research involving human subjects.

Ethics committees are typically required to represent multiple viewpoints and provide mechanisms for inclusiveness^19^. They can serve to motivate moral reflection while demystifying ethics^41^ and can examine complex multi-dimensional issues without reducing them to quantitative metrics^20^. In the social sciences, too, there is a growing reliance on advisory groups composed of people whose expertise spans various domains—academic, policy, lived experience, local knowledge—to co-produce recommendations about, for example, the appropriate understanding of human well-being or thriving for particular policy contexts^42,43^.

Ethics committees to date largely fall into two broad domains^37,41^—those that concern social or biological research (e.g., IRB and IACUC) and those that are intended to limit legislative and political conflicts of interest (implemented at the levels of city, state, and federal offices). We suggest that environmental ethics committees could serve analogous roles, be implemented at various levels of decision-making from international to local scales, and either be part of existing committees or convened on an issue-specific basis. In addition to providing advice to governmental institutions that make and enact environmental policies, they could also play a role in universities and funding agencies in recommending and prioritizing areas of research. Developing clear guidelines for environmental ethics committees would help ensure that they serve a well-defined advisory role, integrate effectively with existing governmental structures, and avoid redundancy or conflict with other institutions. These guidelines should, therefore, establish the committees’ scope, place within a governance framework, and specify their role in decision-making. Guidelines should also be directed towards mitigating conflicts of interest^37^.

Another important role for guidelines would be defining committee membership. A good starting point would be to invite participation from members of the academic, nongovernmental, and public sectors, providing broad perspectives to ensure the protection of ecosystem health alongside human interests^37^. This may include those with climate and ecology expertise, environmental ethicists, social scientists, human rights advocates, and Indigenous or local community leaders. Committees should also include representatives from constituencies that would be most directly affected by a given project^19^, recognizing that impacts may not be spatially localized. Diverse representation on such committees facilitates value-pluralism and awareness of distributional impacts.

There are examples of committees of diverse experts successfully advising on conservation policy at the national level. New Zealand demonstrates leadership that serves as an example. In 2006, the country’s Department of Conservation expanded membership of the Kākāpō Recovery Group (the kākāpō is a critically endangered endemic bird) to include representation of the Ngāi Tahu people, in addition to ecologists and governmental policy experts. The recovery group has overseen a fourfold increase in the kākāpō population, in part due to better collaboration between the Department of Conservation and the Ngāi Tahu^44^. New Zealand is also at the forefront of sub-national conservation research partnerships that follow a similar structure, including a conservation genomics research program for culturally significant freshwater species^45^ and a co-management plan for endemic bat and bird species^46^. Common to all these cases is that committee members brought different, initially conflicting, positions with respect to conservation research and policy to the table and were able to resolve them through an iterative, deliberative process.

Ethics committees may be seen as imposing additional red tape and restrictions on initiatives that are urgently needed to combat both climate change and biodiversity loss. When it comes to local decision-making, participatory governance processes can also be susceptible to NIMBYism (“Not In My Back Yard”), derailing projects with important environmental benefits such as the construction of high-density housing^47^ and siting of renewable energy sources^48,49^. These situations also reinforce historic social inequities through practices such as exclusionary zoning^50^. However, such perverse consequences occur when local constituents disproportionate influence on process outcomes that affect collective welfare, which can be minimized by broadening stakeholder representation. Ethics committees can be implemented in ways that mitigate power imbalances, promoting inclusive, transparent decision-making^19^.

Collaborative governance models do not share a universal blueprint. They are especially fraught when there is a lack of cooperation among actors or when there are mismatches between biophysical structures and governance structures^38^. Different nations, jurisdictions, and communities must develop their own guidelines on how to implement these committees^37^. Diversity of representation is one step in reducing power dynamics, but rotating membership, clearly defined roles and responsibilities, structured speaking time, facilitation training, transparency in deliberation, and periodic structure reviews can also be useful^37^.

## Conclusions

Over-emphasizing win-win outcomes while downplaying trade-offs between climate change mitigation and biodiversity conservation can erode trust and be counterproductive. It is necessary to move beyond simplistic narratives of synergy and develop mechanisms to actually address known trade-offs. To counter over-reliance on monetization and CBAs, we suggest additionally establishing enviro-ethical committees to examine and advise on the varied environmental and human impacts of projects. Such committees would allow disparate perspectives to influence decisions, actively address power imbalances, and provide a participatory mechanism for weighing trade-offs transparently. Of course, doing so may fracture alliances forged by purported synergies. But when such synergies are illusory, we suspect they will fray in any event. Moreover, acknowledging harm to a particular goal (or constituency) increases credibility and trust, creating political pressure to explore compensatory actions. Society has decades of challenging work ahead, and the path forward will not always be obvious or easy. Top-down initiatives are subject to change alongside the priorities of political leaders. By empowering diverse stakeholders and constituencies through bottom-up mechanisms that create a forum for dialogue and deliberation, we can better navigate our journey toward a more equitable and sustainable future.

## Data Availability

No datasets were generated or analysed during the current study.
